# Real-World Treatment Patterns, Clinical Outcomes, and Healthcare Resource Utilization in Early-Stage Non-Small-Cell Lung Cancer

**DOI:** 10.3390/curroncol31010030

**Published:** 2024-01-12

**Authors:** Dylan E. O’Sullivan, Devon J. Boyne, Chelsea Ford-Sahibzada, Jessica A. Inskip, Christopher J. Smith, Kaushik Sripada, Darren R. Brenner, Winson Y. Cheung

**Affiliations:** 1 Department of Oncology, University of Calgary, Calgary, AB T2N 1N4, Canada; dylan.osullivan@ucalgary.ca (D.E.O.); darren.brenner@ucalgary.ca (D.R.B.); 2 Department of Community Health Sciences, University of Calgary, Calgary, AB T2N 1N4, Canada; 3 Oncology Outcomes Initiative, University of Calgary, Calgary, AB T2N 1N4, Canada; 4 Hoffmann-La Roche Limited, Mississauga, ON L5N 5M8, Canada

**Keywords:** early-stage non-small-cell lung cancer, population-based cohort study, real-world data

## Abstract

The prognosis of early non-small-cell lung cancer (eNSCLC) remains poor. An understanding of current therapies and outcomes can provide insights into how novel therapies can be integrated into clinics. We conducted a large, retrospective, population-based cohort study of patients with de novo eNSCLC (stages IB, IIA, IIB, and IIIA) diagnosed in Alberta, Canada, between 2010 and 2019. The primary objectives were to describe treatment patterns and survival outcomes among patients with eNSCLC. A total of 5126 patients with eNSCLC were included. A total of 45.3% of patients were referred to a medical oncologist, ranging from 23.7% in stage IB to 58.3% in IIIA. A total of 23.6% of patients initiated systemic therapy (ST), ranging from 3.5% in stage IB to 38.5% in IIIA. For stage IIB and IIIA individuals who received surgery, adjuvant ST was associated with a decreased likelihood of death (hazard ratios (HR) of 0.77 (95% CI: 0.56–1.07) and 0.69 (95% CI: 0.54–0.89), respectively). In a Canadian real-world setting, stage IIB and IIIA patients who received adjuvant ST tended to have better survival than patients who did not, but future studies that provide adjustment of additional confounders are warranted. Examining referral pathways that account for disparities based on age, sex, and comorbidities in the real world would also provide further insights.

## 1. Introduction

Lung cancer is the most commonly diagnosed cancer in Canada, with an estimated 30,000 (15,000 for both males and females) new cases in 2022; an estimated 1 in 15 Canadians are expected to be diagnosed with lung cancer over the course of their lifetime [1]. Lung cancer is also the leading cause of cancer deaths in Canada, and it is responsible for more cancer deaths among Canadians than the other three major cancer types (breast, colorectal, and prostate) combined [1]. Non-small-cell lung cancer (NSCLC) accounts for approximately 85% of all lung cancer cases [2]. NSCLC is further classified into three distinct histological subtypes: squamous-cell carcinoma, adenocarcinoma, and large-cell carcinoma, of which adenocarcinoma is the most common, comprising around 40% to 43% of all lung cancer cases [2].

Lung cancer survival varies considerably by histology and stage at diagnosis. Historically, most lung cancer cases are diagnosed at advanced stages due in part to the lack of symptoms in early stages and the paucity of screening programs [3,4]. In the locally advanced or metastatic setting (stage IIIB-IV), despite major advances in targeted therapies and immunotherapies, many patients do not receive therapy, and 5-year survival outcomes remain poor [5]. Given the poor survival outcomes associated with late-stage diagnosis, there has been recent evidence in support of screening programs that increase early lung cancer detection [3,4]. Clinical trials of screening programs using low-dose computed tomography (LDCT) in high-risk groups have shown promise in reducing mortality from lung cancer [6,7]. Lung screening programs directed towards high-risk groups now aim to shift the distribution of new lung cancer diagnoses to earlier stages, providing patients with more treatment options and improved survival and quality of life.

Clinical care for patients with eNSCLC is multidisciplinary and can involve a combination of surgery, radiation, and systemic therapy. Evidence-based guidelines are evolving, and systemic therapies used in advanced NSCLC, including immunotherapies [8,9,10,11,12] and targeted therapies, are currently in various stages of development and regulatory approval in eNSCLC [13,14,15]. Over the current study period (2010–2019), no adjuvant or neoadjuvant immunotherapies or targeted therapies, such as tyrosine kinase inhibitors (TKIs), were approved in Canada for NSCLC. Canada has recently received regulatory approval for osimertinib in the adjuvant setting for patients with *EGFR* (epidermal growth factor receptor) mutation-positive eNSCLC (2021) [16]. Regulatory approval for immunotherapies now includes adjuvant atezolizumab (2022) [17], neo-adjuvant nivolumab (2022) [18], and adjuvant pembrolizumab (2023) [19]. Successful implementation and integration of these new therapies, as well as future molecules, will require increased coordination of care between specialties to meaningfully impact patient outcomes.

Given the introduction and broader uptake of lung screening programs and the continued adoption of novel treatments for eNSCLC in the coming years, it is important to characterize current treatment patterns and survival outcomes in this patient population. There are several real-world studies in advanced NSCLC; however, there is a paucity of studies for eNSCLC—particularly in the Canadian setting [5,20]. The objective of this study was to describe the treatment patterns, clinical outcomes, and healthcare resource utilization of patients with eNSCLC in Alberta, Canada.

## 2. Materials and Methods

### 2.1. Study Population

This study was a retrospective longitudinal cohort study of real-world, population-level data in Alberta, Canada. All patients ≥18 years of age who were diagnosed with eNSCLC between 2010 and 2019 were included and followed until the end of 2020. Patients were identified using information from the Alberta Cancer Registry (ACR), and NSCLC stage was defined as the American Joint Committee on Cancer (AJCC) TNM eighth edition stage IB-IIIA. Stage IA patients were excluded, given that these patients are typically not included in clinical trials examining the efficacy of adjuvant or neoadjuvant systemic therapies for eNSCLC. If the eighth edition staging was not available, the AJCC seventh or sixth edition staging was utilized. The staging was not harmonized with the eighth edition. In this study, we primarily relied on pathological staging (90.1%). If a patient was missing pathological stage, we used clinical stage instead (9.9%). All individuals identified and included in this study were linked to other administrative databases using a unique lifetime identifier (ULI) to capture additional variables. The administrative databases included the Electronic Medical Record database, which captures treatment information (surgery, radiation, systemic therapy); the hospital Discharge Abstract Database and the National Ambulatory Care Reporting System database for hospitalizations and emergency room visits; the Practitioner Claims database for physician and practitioner office visits; the Population Registry database for demographic information; and Statistics Canada census data for neighbourhood socioeconomic status measures and place of residence (urban/rural) based on patients’ postal codes at diagnosis. All residents of Alberta are assigned a ULI number, which can be deterministically linked to provincial administrative databases with a 100% linkage rate.

### 2.2. Baseline Patient and Disease Characteristics

Baseline characteristics are presented overall and stratified by receipt of systemic therapy. Characteristics considered in this study included age, sex, stage (IB, IIA, IIB, and IIIA), subtype (non-squamous and squamous), urban/rural residence, neighbourhood-level socioeconomic measures (annual household income and proportion of neighbourhood with at least a high school education), number of Charlson comorbidities, and specific comorbidities (cardiovascular disease, diabetes, chronic obstructive pulmonary disease, liver disease, and renal disease). To compare the distribution of the baseline characteristics between those who initiated systemic therapy and those who did not, *p*-values corresponding to *t*-tests for continuous variables and chi-square tests for categorical variables are presented, as are absolute standardized differences (ASD).

### 2.3. Treatment Patterns

The primary therapies of interest included surgery, systemic therapy (chemotherapy and immunotherapy), and radiation therapy. To ensure that systemic therapy and radiation were for treatment of eNSCLC, we restricted to therapies initiated within one year of diagnosis or within 180 days of surgery for patients who received surgery. The proportions of patients receiving various therapies were calculated overall and by stage (IB, II, and IIIA). With respect to treatment duration, time on therapy was estimated as the time from initiation to the last systemic therapy cycle plus 28 days (patients were censored at death or end of study). Median time on systemic therapy was estimated with the Kaplan–Meier method whereby individuals were censored if they were lost to follow-up prior to the end of systemic therapy.

### 2.4. Survival Outcomes

Overall survival (OS) and cancer-specific survival (CSS) were estimated from the time of diagnosis until death from any cause (OS) or until death due to cancer (CSS). Individuals were censored at the date of last contact with Alberta Health Services or on 31 December 2020, whichever occurred first. Time to event was quantified using the Kaplan–Meier method. Stratified analyses were conducted to select baseline characteristics, including age, sex, stage, comorbidity, surgery, radiation, and systemic therapy. When comparing two or more strata, crude hazard ratios and 95% confidence intervals from a univariable Cox proportional hazard model were provided along with log-rank *p*-values. In addition, we examined the association between systemic therapy use and overall survival using multivariable Cox proportional hazards models while adjusting for important confounders. All variables were specified a priori and included in the model irrespective of statistical significance.

### 2.5. Healthcare Resource Utilization

Healthcare resource utilization (HCRU) was estimated overall and stratified by stage (I/II vs. IIIA) and by receipt of systemic therapy (yes vs. no). HCRU was defined by hospitalization (number of times and number of days), ambulatory care encounters (number of encounters overall, emergency encounters, and non-emergency encounters), cancer physician visits (number of visits overall and categorized by medical oncologist, radiation oncologist, general/family practitioner, other cancer physicians), non-cancer practitioner visits (number of encounters and claims), and number of days of radiation therapy. For each of these outcomes, the mean number of events per patient within each year of follow-up was estimated.

## 3. Results

### 3.1. Baseline Patient and Disease Characteristics

Baseline patient and disease characteristics overall and stratified by receipt of systemic therapy are presented in Table 1. A total of 5146 patients were included in this study. The mean age at diagnosis was 71.3 years (SD = 10.3), and 52.5% were female.

### 3.2. Treatment Patterns

**Figure 1:**
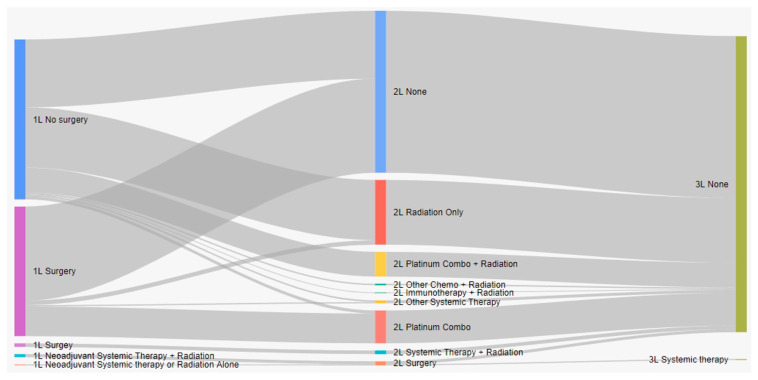
Treatment patterns of patients with eNSCLC in Alberta, Canada (Sankey diagram). 1L: first line; 2L: second line; 3L: third line.

**Figure 2:**
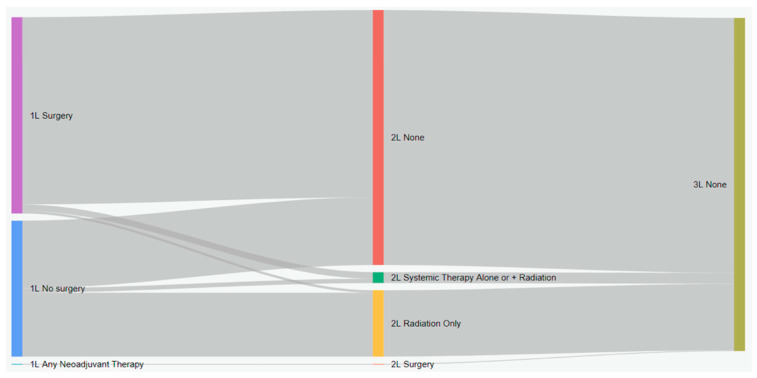
Treatment patterns of patients with stage IB eNSCLC in Alberta, Canada (Sankey diagram). 1L: first line; 2L: second line; 3L: third line.

**Figure 3:**
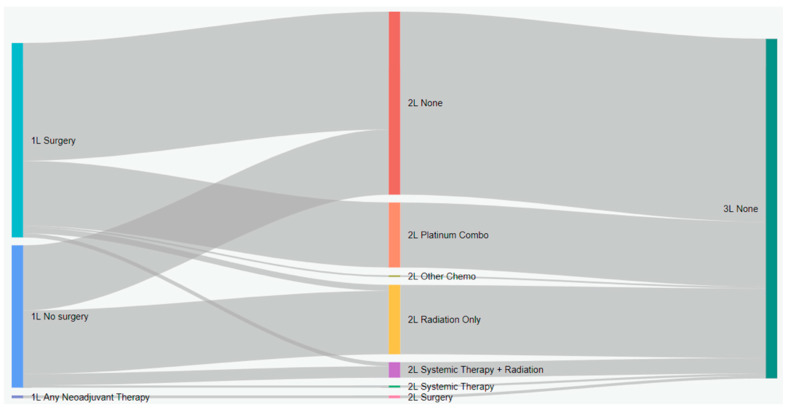
Treatment patterns of patients with stage II eNSCLC in Alberta, Canada (Sankey diagram). 1L: first line; 2L: second line; 3L: third line.

**Figure 4:**
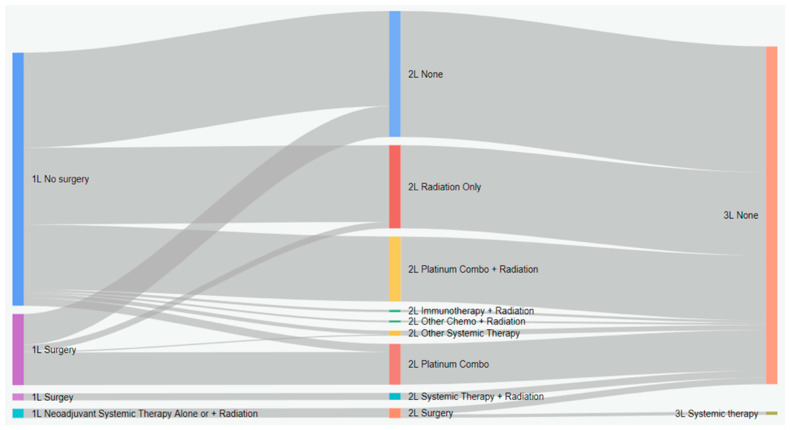
Treatment patterns of patients with stage IIIA eNSCLC in Alberta, Canada (Sankey diagram). 1L: first line; 2L: second line; 3L: third line.

Treatment characteristics of patients with eNSCLC by stage at diagnosis are presented in Table 2. Of 5126 total patients, 2367 (47.2%) received surgery, and 1706 (33.3%) received radiation with surgery (Table 2). Most patients who received surgery as a first treatment did not receive any other treatment (Figure 1). Among all patients who did not have surgery, 19.9% received systemic therapy, and 54.6% received radiation. Nearly all IIIA patients who received neoadjuvant treatment had both systemic and radiation therapy (96.2%). Among patients who received adjuvant treatment (29.5%), most received systemic therapy (88.1%), either alone or in combination with radiation (Figure 1). As the stage increased, the receipt of adjuvant treatment was more likely (*p* < 0.001, SMD = 0.911). Sankey diagrams by stage are provided in Figure 2, Figure 3 and Figure 4.

Among the total cohort, 1210 (23.6%) received systemic therapy. The most common adjuvant regimen and regimen for patients who did not receive surgery was cisplatin plus vinorelbine, while the most common neoadjuvant regimen was cisplatin plus etoposide. Cisplatin plus paclitaxel was the second most common adjuvant regimen and regimen for patients who did not receive surgery. A number of baseline characteristics were associated with receipt of systemic therapy (Table 1). Patients who received systemic therapy were more often under 65 years of age (*p* < 0.001; SMD = 0.523), were male (*p* = 0.02; SMD = 0.079), had a higher stage (*p* < 0.001; SMD = 0.975), and had fewer comorbidities (*p* < 0.001; SMD = 0.375), including less cardiovascular disease (*p* < 0.001; SMD = 0.285), diabetes (*p* < 0.001; SMD = 0.120), chronic obstructive pulmonary disease (*p* = 0.001; SMD = 0.107), and renal disease (*p* < 0.001; SMD = 0.237), respectively. In examining treatment patterns over time, it was found that systemic therapy use among all patients increased from 19.9% in 2010 to 28.0% in 2019 (*p* = 0.03), with the largest absolute increase occurring in patients with stage IIIA (19.5% increase from 29.9% to 49.5%; *p* = 0.003) disease. Of the 5146 patients in this study, 2323 (45.3%) were referred to a medical oncologist, ranging from 23.7% in stage IB and 58.3% in IIIA. Restricting to the 2323 referred individuals, 1118 (50.8%) received systemic therapy, ranging from 13.0% for stage IB to 65.3% for stage IIIA (Table 3).

The median time from diagnosis to systemic therapy was 8.4 weeks for patients who did not receive surgery, 8.9 weeks for patients who received neoadjuvant treatment, and 16.6 weeks for adjuvant systemic therapy. The median time on therapy was 8.0 weeks for patients who did not receive surgery and for patients who received neoadjuvant therapy, while the median time on therapy was 12.3 weeks for patients in the adjuvant setting.

### 3.3. Survival Outcomes

**Figure 5:**
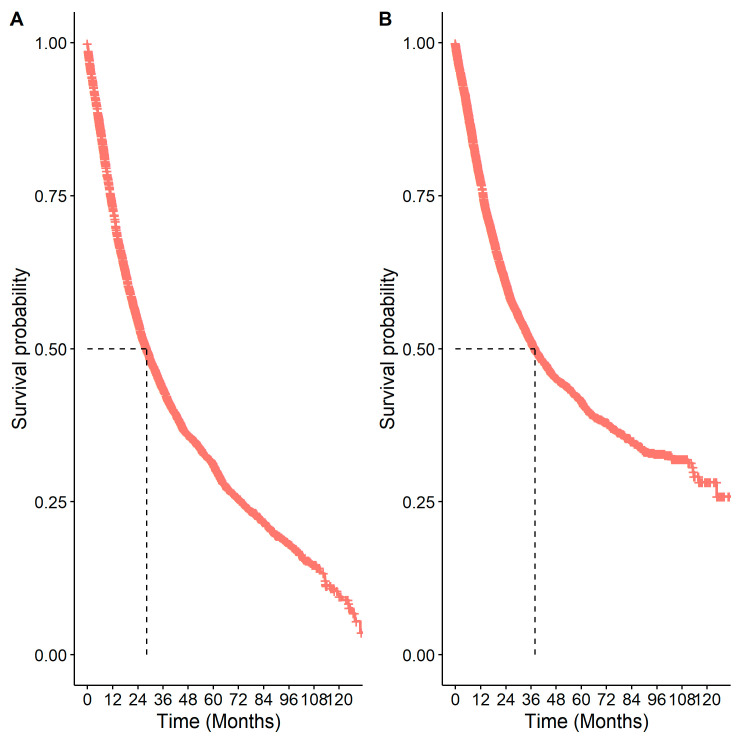
Kaplan–Meier survival curves for patients with eNSCLC in Alberta, Canada: (**A**) overall survival; (**B**) cancer-specific survival. The number of patients at risk at 0 months was 5126.

The median OS from diagnosis was 28.18 months (95% CI: 26.56–29.69), and the median CSS was 37.94 months (95% CI: 35.54–40.90) (Figure 5). The overall 5-year survival was 31.1% (95% CI: 29.7%–32.6%), with a cancer-specific 5-year survival of 41.5% (95% CI: 39.9%–43.2%). There were significant differences in OS by age, sex, comorbidity status, stage, receipt of surgery, systemic therapy, and radiation (*p* < 0.05; Table 4). Patients had reduced survival if they were 65 years or older (HR: 1.84 (1.69–2.00)), had a comorbidity (HR: 1.32 (95% CI: 1.23–1.41)), or were increasing in stage (stage IIA vs. stage IB HR: 1.24 (95% CI: 1.10–1.39); stage IIB vs. stage IB HR: 1.44 (95% CI: 1.30–1.61); stage IIIA vs. stage IB HR: 2.23 (95% CI: 2.04–2.42)). Similar findings were observed for cancer-specific survival and are presented in Supplementary Table S1.

**Figure 6:**
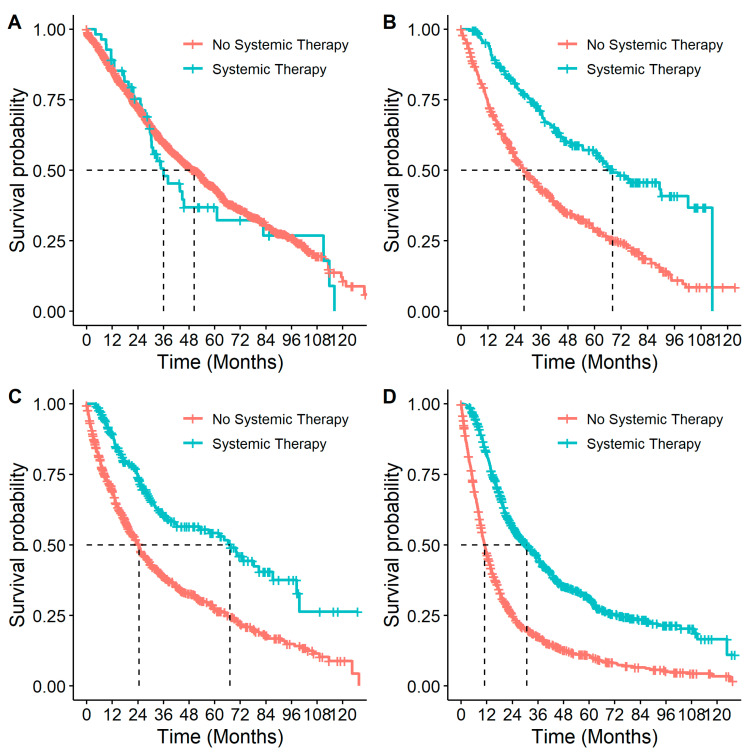
Overall survival in patients with eNSCLC for stage at diagnosis, stratified by treatment with systemic therapy: (**A**) Overall survival of patients diagnosed at stage IB NSCLC by systemic therapy. Number of patients at risk at 0 months was: no systemic therapy *n* = 1535; systemic therapy *n* = 55. (**B**) Overall survival of patients diagnosed at stage IIA NSCLC by systemic therapy. Number of patients at risk at 0 months was: no systemic therapy *n* = 495; systemic therapy *n* = 191. (**C**) Overall survival of patients diagnosed at stage IIB NSCLC by systemic therapy. Number of patients at risk at 0 months was: no systemic therapy *n* = 692; systemic therapy *n* = 217. (**D**) Overall survival of patients diagnosed at stage IIIA NSCLC by systemic therapy. Number of patients at risk at 0 months was: no systemic therapy *n* = 1194; systemic therapy *n* = 747.

**Figure 7:**
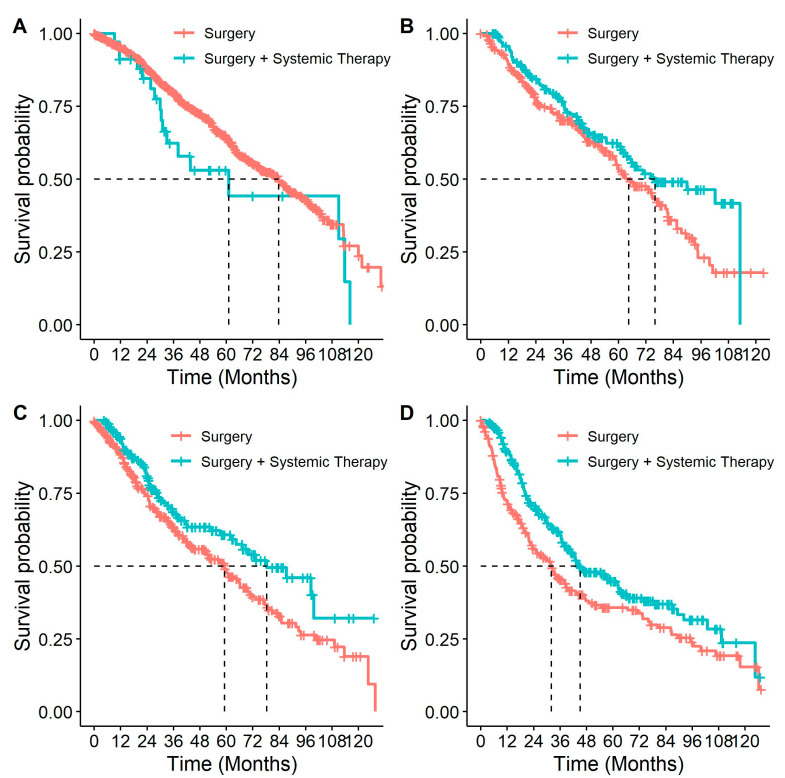
Overall survival in patients with eNSCLC for stage at diagnosis, stratified by patients who received surgery and patients who received surgery & systemic therapy: (**A**) Overall survival of patients diagnosed at stage IB NSCLC, by treatment. Number of patients at risk at 0 months was: surgery *n* = 907; surgery + systemic therapy *n* = 34; (**B**) Overall survival of patients diagnosed at stage IIA NSCLC, by treatment. Number of patients at risk at 0 months was: surgery *n* = 245; surgery + systemic therapy *n* = 167; (**C**) Overall survival of patients diagnosed at stage IIB NSCLC, by treatment. Number of patients at risk at 0 months was: surgery *n* = 338; surgery + systemic therapy *n* = 177; (**D**) Overall survival of patients diagnosed at stage IIIA NSCLC, by treatment. Number of patients at risk at 0 months was: surgery *n* = 215; surgery + systemic therapy *n* = 284.

Patients who received systemic therapy had better OS from diagnosis than those who did not (*p* < 0.001, HR: 0.69 (0.63–0.75)). When this association was examined by stage (Figure 6), it remained significant for all but stage IB patients (*p* = 0.40, Table 4). Similar to stratifying by systemic therapy, stratifying by receipt of systemic therapy and surgery compared to surgery alone found differences in survival among stage II and IIIA patients, with better survival among patients who had received both surgery and systemic therapy compared to patients who only received surgery (Table 4 and Figure 7). In multivariable analyses, for stage IIB and IIIA individuals who received surgery, adjuvant systemic therapy was also associated with a decreased likelihood of death with HRs of 0.77 (95% CI: 0.56–1.07) and 0.69 (95% CI: 0.54–0.89), respectively.

### 3.4. Healthcare Resource Utilization

The mean healthcare resource utilization per patient per year is presented in Table 5. Overall, average healthcare resource utilization decreased considerably after the first year and decreased slightly each year after. Healthcare resource utilization by stage and receipt of systemic therapy are presented in Supplementary Tables S2–S5. Patients who initiated systemic therapy had more cancer physician visits but fewer hospitalizations and ambulatory care visits in the first year after diagnosis compared to patients who did not initiate systemic therapy.

## 4. Discussion

In this study, we evaluated the treatment patterns, survival outcomes, and healthcare resource utilization among a cohort of real-world patients with eNSCLC in the province of Alberta, Canada. There were several notable findings within our results. Fewer patients received surgery than predicted, which was especially high in stage IIIA, where three-quarters of patients did not receive surgery. Referral to a medical oncologist also occurred at a lower rate than expected, with less than half of all patients being referred. Even when patients were referred to an oncologist, the use of systemic therapy could be considered low among those with stage II and IIIA disease.

Within this real-world population, 47.2% of the individuals received surgical treatment, which is comparable to treatment patterns seen in previous research [20]. Arnold et al. and Pinquie et al. observed that resection occurred in 38.1% and 50.3% of their study cohorts, respectively [21,22]. The decrease that we observed in the proportion of patients receiving surgery as stage increases, with 59.2% of stage IB patients receiving surgery compared to 25.7% of stage IIIA patients, has also been previously reported. Studies examining surgery have found that the proportions of stage I NSCLC patients receiving surgery range from 68.2% to 78.6%, while in stage III patients, the proportions drop to between 10.3% and 17.5% [21,22,23,24]. We observed a larger-than-predicted proportion of patients with eNSCLC who did not undergo surgery. While it has been clearly demonstrated that surgical treatment offers a survival advantage in eNSCLC, this population of non-surgical intervention patients highlights the importance of systemic therapy within the disease [25,26].

Less than 25% of this population was reported to have received systemic therapy, with those who had more advanced cancer stages and fewer comorbidities having an increased likelihood of receipt. Similar usage of systemic therapy has been demonstrated in comparable populations, with systemic therapy receipt reported between 24.2% and 28.3% [21,27,28]. Neoadjuvant systemic therapy was rare and restricted to later-stage patients, which has been reported in other studies [29,30]. While adjuvant and standalone systemic therapy were used more often than the neoadjuvant regimen, systemic therapy as a whole may not be utilized as frequently as may be expected or recommended [24,31]. There has been a significant increase in systemic therapy use over time, which has also been reported in other studies [20].

When stratified by stage, patients with stage II and IIIA disease had significantly better survival when receiving systemic therapy and surgery compared to surgery alone. The finding that surgical patients receiving systemic therapy have better survival than their surgery-only counterparts is an outcome that has been reported with increasing frequency [32,33,34,35]. However, the use of systemic therapy is impacted by socioeconomic disparities and other barriers that warrant additional study to ensure all patients have the opportunity to access new treatment options that are expected to further improve survival [36].

Targeted and immunotherapy treatments that can be specifically selected on the basis of molecular markers are now widely used in advanced NSCLC, and trials of these agents are reading out in the adjuvant and neoadjuvant settings [37,38]. As demonstrated in this study, systemic therapy has thus far been largely restricted to adjuvant treatment with chemotherapy, but the adoption of these new targeted and immunotherapy treatments in eNSCLC could facilitate progress in the adjuvant and neoadjuvant setting as well.

Given the potential survival benefit of systemic therapy in eNSCLC, it is important to examine the factors that may contribute to the best utilization of these treatments. One potential component that was demonstrated within this study was the referral rate of patients to medical oncologists. There are sparse data on medical oncologist referral rates in the literature, but an older local Alberta-based study found that 79% of non-surgical and 72% of surgical stage IB to IIB eNSCLC patients had a consultation with a medical oncologist [39]. We observed a considerably lower referral rate of 45.3%, likely due to the population-based nature of this study. Even when patients were referred to an oncologist, half of the referrals resulted in the receipt of systemic treatment.

With respect to HCRU, the number of hospitalizations and ambulatory care encounters among individuals with eNSCLC was comparable to that of individuals with extensive-stage small-cell lung cancer and *EGFR*-positive metastatic NSCLC reported in prior investigations [40,41]. In contrast, individuals with eNSCLC tended to have fewer medical oncology visits, fewer cycles of chemotherapy, and a greater number of non-cancer practitioner claims. Treatment-related differences may account for these disparities in HCRU.

To our knowledge, this is one of the first studies to estimate healthcare resource utilization in the eNSCLC setting in Canada. The highest resource usage was in the first year, likely when patients were receiving treatment but also when relapses were likely to occur. Patients who initiated systemic therapy had less non-treatment-related healthcare utilization compared with patients not treated with systemic therapy but higher use related to treatment. Emerging therapies for this patient population may also impact healthcare resource utilization by reducing downstream health costs.

Our investigation has notable strengths. First, we relied on population-level data, which captures all individuals with a diagnosis of eNSCLC in Alberta and allows for the accurate identification of referral and treatment patterns. Other strengths include high-quality data on systemic therapies, little missing data on captured variables, and a short lag period between the current calendar date and the end of follow-up. Lastly, this study was conducted over a 10-year period and represents one of the largest studies to date on this patient population.

This study has limitations. Driver mutation status or programmed death-protein ligand 1 (PD-L1) expression was not described as these data were collected prior to when testing for these markers in eNSCLC was commonplace. This study utilized administrative data, which do not routinely capture some important variables such as lifestyle factors, performance status or toxicity from treatment. While we conducted multivariable analyses for the association of receipt of systemic therapy with survival, we were unable to adjust for all important covariates and did not take into account immortal time or other sources of bias. Therefore, this was an association and not an estimate of a causal effect and does not reflect the comparative effectiveness of systemic therapy.

## 5. Conclusions

In a Canadian real-world setting, patients with stage IIB and IIIA NSCLC who received adjuvant systemic therapy tended to have better survival than patients who did not, but future studies that provide adjustment of additional potential confounders are warranted. While the rate of systemic therapy use has increased over time in this patient population, a considerable proportion of patients are not referred to a medical oncologist to be considered for systemic therapy. Our findings underscore the importance of optimizing referrals to enable the timely initiation of appropriate therapies for patients with lung cancer in order to improve their outcomes. An example of this put into action includes the Alberta Thoracic Oncology Program [42], which aims to streamline the diagnostic and referral pathways and facilitate more immediate access to consulting physicians to minimize the delays posed by poor healthcare system navigation. Further work to develop and enhance referral pathways appears to be an important step to ensure that emerging novel therapies are integrated effectively into the real world so that potential survival gains from new drugs can be realized. It will be important for such referral pathways to account for disparities based on age, sex, and comorbidities in the real world.

## Figures and Tables

**Table 1:** Baseline patient and disease characteristics of patients with eNSCLC in Alberta, Canada.

Variable	Overall (*n* = 5126)	Systemic Therapy (*n* = 1210)	No Systemic Therapy (*n* = 3916)	*p*-Value	SMD
Age, years (mean (SD))	71.3 (10.3)	65.5 (8.7)	73.0 (10.1)	<0.001	0.79
Age (*n* (%))					
<65 years	1285 (25.1)	520 (43.0)	765 (19.5)	<0.001	0.523
65+ years	3841 (74.9)	690 (57.0)	3151 (80.5)		
Sex (*n* (%))				0.02	0.079
Male	2433 (47.5)	611 (50.5)	1822 (46.5)		
Female	2693 (52.5)	599 (49.5)	2094 (53.5)		
AJCC Stage (*n* (%))				<0.001	0.975
IB	1590 (31.0)	55 (4.6)	1535 (39.2)		
IIA	686 (13.4)	191 (15.8)	495 (12.6)		
IIB	909 (17.7)	217 (17.9)	692 (17.7)		
IIIA	1941 (37.9)	747 (61.7)	1194 (30.5)		
Subtype (*n* (%))				0.98	0.002
Squamous	1560 (30.4)	369 (30.5)	1191 (30.4)		
Non-squamous	3566 (69.6)	841 (69.5)	2725 (69.6)		
Categories of Neighbourhood Annual Household Income (*n* (%))				0.18	0.076
0–25 k	203 (4.0)	35 (2.9)	168 (4.3)		
25–35 k	942 (18.4)	225 (18.6)	717 (18.3)		
35–45	1708 (33.3)	404 (33.4)	1304 (33.3)		
45 k+	2265 (44.2)	545 (45.0)	1720 (43.9)		
Missing	8 (0.2)	1 (0.1)	7 (0.2)		
Categories of Neighbourhood Education (*n* (%))				0.06	0.092
0.00–0.60	363 (7.1)	66 (5.5)	297 (7.6)		
0.60–0.70	733 (14.3)	169 (14.0)	564 (14.4)		
0.70–0.80	1391 (27.1)	345 (28.5)	1046 (26.7)		
0.80+	2631 (51.3)	629 (52.0)	2002 (51.1)		
Missing	8 (0.2)	1 (0.1)	7 (0.2)		
Charlson Comorbidity Index (*n* (%))				<0.001	0.375
0	2169 (42.3)	615 (50.8)	1554 (39.7)		
1	1665 (32.5)	420 (34.7)	1245 (31.8)		
2	681 (13.3)	109 (9.0)	572 (14.6)		
3	327 (6.4)	44 (3.6)	283 (7.2)		
4+	284 (5.5)	22 (1.8)	262 (6.7)		
Cardiovascular disease (*n* (%)) ^1^	838 (16.3)	108 (8.9)	730 (18.6)	<0.001	0.285
Diabetes (*n* (%))	981 (19.1)	189 (15.6)	792 (20.2)	<0.001	0.120
Chronic Obstructive Pulmonary Disease (*n* (%))	1908 (37.2)	403 (33.3)	1505 (38.4)	0.001	0.107
Connective Tissue Disease (*n* (%))	112 (2.2)	21 (1.7)	91 (2.3)	0.26	0.042
Liver disease (*n* (%))	95 (1.9)	24 (2.0)	71 (1.8)	0.79	0.012
Renal disease (*n* (%))	230 (4.5)	15 (1.2)	215 (5.5)	<0.001	0.237

SMD: standardized mean difference. COPD: chronic obstructive pulmonary disease. ^1^ Includes congestive heart failure, myocardial infarction, peripheral vascular disease, and cerebrovascular disease (assessed within six months of diagnosis).

**Table 2:** Treatment characteristics of patients with eNSCLC by stage at diagnosis.

Variable	Overall (*n* = 5126)	IB (*n* = 1590)	II (*n* = 1595)	IIIA (*n* = 1941)	*p*-Value	SMD
Surgery (%)	2367 (47.2)	941 (59.2)	927 (58.1)	499 (25.7)	<0.001	0.479
Systemic therapy (%)	1210 (23.6)	55 (3.5)	408 (25.6)	747 (38.5)	<0.001	0.631
Radiation therapy (%)	1706 (33.3)	338 (21.3)	410 (25.7)	958 (49.4)	<0.001	0.408
**No Surgery (%)**	2759 (53.8)	649 (40.8)	668 (41.9)	1442 (74.3)	<0.001	0.479
Systemic therapy	548 (19.9)	21 (3.2)	64 (9.6)	463 (32.1)	<0.001	0.552
Radiation therapy	1506 (54.6)	322 (49.6)	352 (52.7)	832 (57.7)	0.002	0.108
**Neoadjuvant ^1^ (%)**	71 (3.0)	<10	14 (1.5)	53 (10.6)	<0.001	0.319
Systemic therapy	66 (2.8)	<10	12 (1.3)	51 (10.2)	<0.001	0.318
Radiation therapy	58 (2.5)	<10	11 (1.2)	46 (9.2)	<0.001	0.315
**Adjuvant ^2^ (%)**	699 (29.5)	44 (4.7)	363 (39.2)	292 (58.5)	<0.001	0.911
Systemic therapy	616 (26.0)	31 (3.3)	333 (35.9)	254 (50.9)	<0.001	0.819
Radiation therapy	150 (6.3)	16 (1.7)	50 (5.4)	84 (16.8)	<0.001	0.370

SMD: standardized mean difference. ^1^ Systemic therapy or radiation therapy after diagnosis and prior to surgery. ^2^ Systemic therapy or radiation therapy within 180 days of surgery.

**Table 3:** Referral to medical oncologist and systemic therapy among referred patients.

Variable	Overall (*n* = 5126)	IB (*n* = 1590)	IIA (*n* = 1595)	IIIA (*n* = 1941)
**Referred to Medical Oncologist (*n* [%])**	2323 (45.3)	377 (23.7)	815 (51.1)	1131 (58.3)
**Systemic Therapy among** **Referred Patients (*n* [%])**	1181 (50.8)	49 (13.0)	394 (48.3)	738 (65.3)

**Table 4:** Overall survival, log-rank tests, and crude hazard ratios for each subgroup analysis.

Variable	Strata	Median Survival ^1^ (95% CI)	1-Year Survival (95% CI)	2-Year Survival (95% CI)	5-Year Survival (95% CI)	Log-Rank Test *p*-Value	Crude Hazard Ratio (95% CI)
Age	<65 years	54.74 (45.60–60.30)	0.829 (0.809–0.851)	0.676 (0.650–0.704)	0.472 (0.441–0.505)	<0.001	Ref (1.0)
	≥65 years	24.26 (23.08–25.35)	0.698 (0.684–0.713)	0.503 (0.487–0.520)	0.258 (0.243–0.275)		1.84 (1.69–2.00)
Sex	Male	23.84 (22.45–22.15)	0.699 (0.681–0.718)	0.498 (0.478–0.519)	0.272 (0.252–0.293)	<0.001	Ref (1.0)
	Female	33.11 (31.00–35.54)	0.760 (0.744–0.777)	0.591 (0.572–0.610)	0.347 (0.327–0.369)		0.80 (0.74–0.85)
Comorbidity	No	35.31 (32.71–37.97)	0.797 (0.780–0.815)	0.601 (0.580–0.623)	0.361 (0.338–0.386)	<0.001	Ref (1.0)
	Yes	24.49 (22.95–25.91)	0.683 (0.666–0.700)	0.507 (0.489–0.526)	0.276 (0.258–0.295)		1.32 (1.23–1.41)
Stage	IB	49.01 (45.00–54.15)	0.855 (0.837–0.873)	0.724 (0.702–0.748)	0.435 (0.407–0.465)	<0.001	Ref (1.0)
	IIA	36.56 (32.94–42.25)	0.798 (0.768–0.829)	0.626 (0.590–0.665)	0.369 (0.331–0.412)		1.24 (1.10–1.39)
	IIB	29.23 (25.32–33.11)	0.741 (0.712–0.770)	0.561 (0.528–0.596)	0.333 (0.298–0.372)		1.44 (1.30–1.61)
	IIIA	16.50 (15.39–17.59)	0.605 (0.584–0.628)	0.373 (0.352–0.396)	0.186 (0.167–0.206)		2.23 (2.04–2.42)
Systemic therapy	No	25.15 (24.07–26.70)	0.691 (0.677–0.706)	0.517 (0.501–0.533)	0.287 (0.271–0.304)	<0.001	Ref (1.0)
	Yes	37.51 (35.44–42.21)	0.857 (0.837–0.877)	0.641 (0.613–0.669)	0.389 (0.358–0.422)		0.69 (0.63–0.75)
Surgery	No	15.29 (14.43–16.34)	0.587 (0.569–0.606)	0.352 (0.334–0.370)	0.121 (0.107–0.136)	<0.001	Ref (1.0)
	Yes	66.61 (63.45–73.32)	0.907 (0.895–0.919)	0.790 (0.773–0.808)	0.553 (0.529–0.578)		0.27 (0.25–0.29)
Radiation therapy	No	36.13 (33.27–38.37)	0.752 (0.737–0.767)	0.601 (0.584–0.619)	0.379 (0.361–0.399)	<0.001	Ref (1.0)
	Yes	20.52 (19.00–22.12)	0.692 (0.670–0.714)	0.445 (0.421–0.469)	0.181 (0.161–0.204)		1.60 (1.49–1.71)
Systemic therapy in stage IB	No	50.43 (45.53–54.67)	0.854 (0.836–0.872)	0.723 (0.700–0.747)	0.437 (0.409–0.468)	0.4	Ref (1.0)
	Yes	36.00 (30.21–82.85)	0.891 (0.812–0.977)	0.754 (0.646–0.880)	0.368 (0.247–0.550)		1.16 (0.82–1.63)
Systemic therapy in stage IIA	No	28.31 (24.26–34.29)	0.738 (0.700–0.779)	0.555 (0.512–0.602)	0.293 (0.251–0.342)	<0.001	Ref (1.0)
	Yes	68.15 (54.74-NA)	0.952 (0.921–0.983)	0.808 (0.752–0.867)	0.562 (0.489–0.646)		0.44 (0.35–0.56)
Systemic therapy in stage IIB	No	24.49 (21.37–27.16)	0.694 (0.660–0.730)	0.505 (0.467–0.546)	0.274 (0.237–0.317)	<0.001	Ref (1.0)
	Yes	67.17 (42.21–98.37)	0.887 (0.844–0.931)	0.740 (0.680–0.806)	0.543 (0.467–0.631)		0.47 (0.38–0.60)
Systemic therapy in stage IIIA	No	10.98 (10.16–11.97)	0.469 (0.442–0.499)	0.253 (0.229–0.280)	0.109 (0.091–0.130)	<0.001	Ref (1.0)
	Yes	30.81 (26.47–35.44)	0.822 (0.794–0.850)	0.564 (0.529–0.602)	0.310 (0.274–0.350)		0.43 (0.38–0.48)
Surgery and systemic therapy	Surgery only	68.91 (63.88–74.93)	0.901 (0.887–0.916)	0.796 (0.776–0.817)	0.561 (0.533–0.590)	0.5	Ref (1.0)
	Both	63.48 (57.07–74.10)	0.920 (0.900–0.942)	0.775 (0.742–0.809)	0.532 (0.490–0.578)		1.05 (0.96–1.20)
Surgery and systemic therapy in stage IB	Surgery only	83.70 (74.33–92.94)	0.952 (0.938–0.967)	0.878 (0.855–0.901)	0.645 (0.608–0.685)	0.08	Ref (1.0)
	Both	61.12 (32.98-NA)	0.912 (0.821–1.000)	0.845 (0.729–0.980)	0.530 (0.368–0.763)		1.56 (0.95–2.55)
Surgery and systemic therapy in stage IIA	Surgery only	64.4 (58.72–80.48)	0.893 (0.854–0.933)	0.777 (0.725–0.834)	0.542 (0.473–0.622)	0.04	Ref (1.0)
	Both	75.91 (63.45-NA)	0.957 (0.926–0.989)	0.842 (0.787–0.901)	0.613 (0.536–0.701)		0.73 (0.54–0.99)
Surgery and systemic therapy in stage IIB	Surgery only	59.15 (45.07–69.67)	0.887 (0.852–0.923)	0.745 (0.696–0.798)	0.484 (0.422–0.555)	0.01	Ref (1.0)
	Both	78.35 (64.37-NA)	0.932 (0.894–0.972)	0.810 (0.749–0.877)	0.608 (0.523–0.705)		0.69 (0.51–0.93)
Surgery and systemic therapy in stage IIIA	Surgery only	32.09 (23.51–39.06)	0.724 (0.665–0.787)	0.562 (0.497–0.635)	0.358 (0.293–0.438)	0.002	Ref (1.0)
	Both	45.17 (40.04–62.60)	0.893 (0.857–0.931)	0.707 (0.653–0.764)	0.449 (0.388–0.520)		0.70 (0.55–0.88)

Ref: reference group for the subgroup hazard ratio analysis; CI: confidence interval; NA: too few deaths to estimate confidence interval. ^1^ Median survival in months.

**Table 5:** Overall mean healthcare resource utilization per patient per year, calculated from all patients with eNSCLC from diagnosis.

Construct	Outcome	Year 1 (*n* = 5126)	Year 2 (*n* = 3524)	Year 3 (*n* = 2397)	Year 4 (*n* = 1668)	Year 5 (*n* = 1129)
Hospitalizations	No. of Hospitalizations	1.39	0.67	0.51	0.49	0.46
	No. of Days Hospitalized	13.52	8.27	6.26	5.25	5.01
Ambulatory Care Services	No. of Encounters	11.89	7.58	6.92	6.44	6.15
	No. of Emergency Encounters	1.98	1.54	1.35	1.2	1.23
	No. of Non-emergency Encounters	9.91	6.04	5.57	5.25	4.92
Cancer Physician Visits	No. of Visits	4.68	2.58	2.18	1.92	1.73
	No. of Medical Oncologist Visits	2.19	1.5	1.37	1.2	1.16
	No. of Radiation Oncologist Visits	2.1	0.84	0.64	0.58	0.45
	No. of General/Family Practitioner Visits	0.13	0.08	0.05	0.04	0.03
	No. of Other Cancer Physician Visits	0.26	0.17	0.12	0.11	0.09
Non-Cancer Practitioner Visits	No. of Encounters	33.62	23.91	21.47	19.95	20.79
	No. of Claims	68.11	41.84	36.98	34.76	35.87
	Final Claims Assessment Amount	7790.71	3456.5	2918.52	2719.26	2838.39
Radiation Therapy	No. of Days of Therapy	7.97	1.18	0.92	0.78	0.62
Chemotherapy Cycles	No. of Cycles	1.72	0.78	0.69	0.67	0.6

## Data Availability

Aggregate-level data presented in this study are available on request from the corresponding author. Individual-level data are not publicly available due to Canadian data privacy laws governing personal health information.

## Supplementary Materials

The following supporting information can be downloaded at https://www.mdpi.com/article/10.3390/curroncol31010030/s1. Table S1: Cancer-specific survival, log-rank tests, and crude hazard ratios for each subgroup analysis; Table S2: Mean healthcare resource utilization per patient per year, calculated from diagnosis, among eNSCLC patients with stage I or II diseases; Table S3: Mean healthcare resource utilization per patient per year, calculated from diagnosis, among eNSCLC patients with stage IIIA disease; Table S4: Mean healthcare resource utilization per patient per year, calculated from diagnosis, among eNSCLC patients who received systemic therapy; Table S5: Mean healthcare resource utilization per patient per year, calculated from diagnosis, among eNSCLC patients who did not receive systemic therapy.
