# Supplementary material for: Real-World Treatment Patterns, Clinical Outcomes, and Healthcare Resource Utilization in Early-Stage Non-Small-Cell Lung Cancer
Source: Curr Oncol. 2024 Jan 12;31(1):447–61. doi: 10.3390/curroncol31010030 (PMC10814046; doi:10.3390/curroncol31010030)
Supplement: Supplementary file 1 [file curroncol-31-00030-s001.zip › curroncol-2726033-supplementary.pdf]

Table S1. Cancer-specific survival, log-rank tests, and crude hazard ratios for each subgroup analysis.

Variable	Strata	Median Survival[1] (95% CI)	1-Year Survival (95% CI)	2-Year Survival (95% CI)	5-Year Survival (95% CI)	Log-rank Test P-value	Crude Hazard Ratio (95% CI)
Age	<65 years	83.70 (70.78-NA)	0.857 (0.838-0.877)	0.715 (0.689-0.742)	0.561 (0.530-0.594)	<0.001	Ref (1.0)
	>=65 years	31.96 (29.65-34.42)	0.744 (0.730-0.759)	0.572 (0.556-0.589)	0.363 (0.345-0.383)		1.74 (1.57-1.92)
Sex	Male	31.00 (27.68-34.59)	0.746 (0.728-0.764)	0.564 (0.543-0.585)	0.378 (0.356-0.403)	<0.001	Ref (1.0)
	Female	45.50 (41.29-51.48)	0.797 (0.782-0.813)	0.649 (0.630-0.668)	0.448 (0.425-0.471)		0.81 (0.75-0.87)
Comorbidity	No	44.55 (40.34-52.70)	0.821 (0.805-0.838)	0.646 (0.625-0.668)	0.451 (0.426-0.478)	<0.001	Ref (1.0)
	Yes	33.24 (30.81-36.49)	0.737 (0.721-0.754)	0.581 (0.562-0.600)	0.388 (0.367-0.410)		1.24 (1.14-1.34)
Stage	IB	89.23 (77.19-NA)	0.900 (0.884-0.915)	0.797 (0.776-0.819)	0.577 (0.547-0.609)	<0.001	Ref (1.0)
	IIA	58.88 (44.65-74.93)	0.831 (0.802-0.860)	0.685 (0.650-0.723)	0.497 (0.455-0.542)		1.43 (1.24-1.64)
	IIB	41.06 (33.80-57.60)	0.788 (0.761-0.816)	0.628 (0.594-0.663)	0.443 (0.404-0.486)		1.70 (1.49-1.93)
	IIIA	18.84 (17.75-20.05)	0.645 (0.623-0.667)	0.423 (0.400-0.447)	0.246 (0.224-0.270)		2.98 (2.69-3.30)
Systemic therapy	No	34.59 (32.15-37.94)	0.738 (0.724-0.752)	0.585 (0.569-0.602)	0.397 (0.378-0.416)	<0.001	Ref (1.0)
	Yes	48.76 (42.71-60.92)	0.881 (0.863-0.900)	0.681 (0.654-0.710)	0.471 (0.438-0.505)		0.74 (0.67-0.81)
Surgery	No	18.64 (17.49-19.60)	0.642 (0.623-0.660)	0.418 (0.398-0.438)	0.197 (0.178-0.217)	<0.001	Ref (1.0)
	Yes	113.79 (113.00-NA)	0.928 (0.917-0.939)	0.834 (0.818-0.850)	0.662 (0.639-0.685)		0.24 (0.22-0.26)
Radiation therapy	No	55.33 (49.22-61.71)	0.795 (0.781-0.809)	0.665 (0.648-0.682)	0.487 (0.467-0.508)	<0.001	Ref (1.0)
	Yes	24.13 (22.98-25.78)	0.731 (0.710-0.753)	0.503 (0.479-0.529)	0.271 (0.245-0.299)		1.65 (1.52-1.78)
Systemic therapy in stage IB	No	89.23 (77.19-NA)	0.899 (0.883-0.915)	0.796 (0.775-0.818)	0.578 (0.547-0.611)	0.3	Ref (1.0)
	Yes	61.12 (34.68-NA)	0.926 (0.859-0.998)	0.823 (0.725-0.936)	0.545 (0.405-0.732)		1.27 (0.84-1.94)

**Systemic therapy in stage IIA**	**No**	39.98 (34.29-50.60)	0.776 (0.739-0.815)	0.624 (0.581-0.671)	0.431 (0.382-0.486)	<0.001	Ref (1.0)
	**Yes**	113.00 (89.33-NA)	0.967 (0.942-0.993)	0.837 (0.784-0.893)	0.655 (0.583-0.736)		0.45 (0.34-0.60)
**Systemic therapy in stage IIB**	**No**	32.61 (27.16-39.12)	0.745 (0.712-0.779)	0.579 (0.540-0.621)	0.380 (0.336-0.429)	<0.001	Ref (1.0)
	**Yes**	NA (87.25-NA)	0.920 (0.883-0.958)	0.779 (0.721-0.843)	0.652 (0.579-0.734)		0.43 (0.33-0.57)
**Systemic therapy in stage IIIA**	**No**	12.56 (11.44-13.84)	0.514 (0.485-0.544)	0.302 (0.275-0.331)	0.161 (0.137-0.188)	<0.001	Ref (1.0)
	**Yes**	36.33 (32.12-41.46)	0.845 (0.819-0.872)	0.605 (0.570-0.643)	0.375 (0.336-0.419)		0.43 (0.38-0.48)
**Surgery and systemic therapy**	**Surgery only**	NA (113.29-NA)	0.922 (0.908-0.935)	0.844 (0.826-0.863)	0.684 (0.657-0.712)	0.004	Ref (1.0)
	**Both**	102.90 (87.26-NA)	0.944 (0.926-0.962)	0.807 (0.776-0.840)	0.607 (0.564-0.653)		1.27 (1.08-1.49)
**Surgery and systemic therapy in stage IB**	**Surgery only**	NA (113.29-NA)	0.969 (0.957-0.981)	0.916 (0.897-0.936)	0.766 (0.731-0.803)	0.001	Ref (1.0)
	**Both**	61.12 (38.07-NA)	0.941 (0.865-1.000)	0.873 (0.763-0.998)	0.576 (0.408-0.813)		2.40 (1.39-4.15)
**Surgery and systemic therapy in stage IIA**	**Surgery only**	94.59 (80.48-NA)	0.909 (0.872-0.947)	0.818 (0.768-0.871)	0.685 (0.620-0.757)	0.2	Ref (1.0)
	**Both**	113.00 (NA-NA)	0.975 (0.951-0.999)	0.871 (0.819-0.925)	0.709 (0.635-0.791)		0.79 (95% CI: 0.54-1.14)
**Surgery and systemic therapy in stage IIB**	**Surgery only**	83.31 (70.78-NA)	0.908 (0.877-0.941)	0.813 (0.768-0.860)	0.610 (0.546-0.681)	0.05	Ref (1.0)
	**Both**	NA (NA-NA)	0.957 (0.926-0.989)	0.832 (0.772-0.896)	0.713 (0.635-0.801)		0.69 (95% CI: 0.47-1.00)
**Surgery and systemic therapy in stage IIIA**	**Surgery only**	43.04 (31.69-76.04)	0.758 (0.701-0.820)	0.619 (0.554-0.693)	0.449 (0.377-0.534)	0.03	Ref (1.0)
	**Both**	58.82 (43.69-92.22)	0.918 (0.885-0.951)	0.747 (0.695-0.803)	0.500 (0.436-0.573)		0.75 (95% CI: 0.58-0.97)

Ref: reference group for the subgroup hazard ratio analysis; CI: confidence interval; NA: too few deaths to estimate confidence interval.

[1] Median survival in months.

Table S2. Mean healthcare resource utilization per patient per year, calculated from diagnosis, among eNSCLC patients with stage I or II disease.

Construct	Outcome	Year 1 (n=3185)	Year 2 (n=2382)	Year 3 (n=1750)	Year 4 (n=1230)	Year 5 (n=857)
**Hospitalizations**	**No. of Hospitalizations**	1.43	0.63	0.5	0.49	0.47
	**No. of Days Hospitalized**	13.24	7.46	6.15	5.12	5.35
**Ambulatory Care Services**	**No. of Encounters**	12.34	7.96	7.08	6.72	6.2
	**No. of Emergency Encounters**	1.8	1.47	1.37	1.19	1.27
	**No. of Non-emergency Encounters**	10.53	6.49	5.72	5.53	4.93
**Cancer Physician Visits**	**No. of Visits**	3.05	1.84	1.64	1.46	1.26
	**No. of Medical Oncologist Visits**	1.35	0.94	0.92	0.81	0.76
	**No. of Radiation Oncologist Visits**	1.41	0.71	0.56	0.52	0.41
	**No. of General/Family Practitioner Visits**	0.08	0.05	0.06	0.03	0.02
	**No. of Other Cancer Physician Visits**	0.21	0.13	0.1	0.1	0.07
**Non-cancer Practitioner Visits**	**No. of Encounters**	34.33	23.95	21.81	20.5	21.22
	**No. of Claims**	70.19	41.38	37.38	35.43	36.66
	**Final Claims Assessment Amount**	8502.06	3494.62	2964.74	2767.04	2859.98
**Radiation Therapy**	**No. of Days of Therapy**	4.51	0.95	0.81	0.7	0.7

**Chemotherapy Cycles**	**No. of Cycles**	0.96	0.49	0.49	0.47	0.47

Table S3. Mean healthcare resource utilization per patient per year, calculated from diagnosis, among eNSCLC patients with stage IIIA disease.

**Construct**	**Outcome**	**Year 1 (n=1941)**	**Year 2 (n=1142)**	**Year 3 (n=647)**	**Year 4 (n=438)**	**Year 5 (n=272)**
**Hospitalizations**	**No. of Hospitalizations**	1.31	0.77	0.54	0.48	0.41
	**No. of Days Hospitalized**	13.98	9.94	6.55	5.64	3.95
**Ambulatory Care Services**	**No. of Encounters**	11.17	6.79	6.49	5.66	6
	**No. of Emergency Encounters**	2.28	1.7	1.31	1.21	1.1
	**No. of Non-emergency Encounters**	8.89	5.09	5.18	4.45	4.9
**Cancer Physician Visits**	**No. of Visits**	7.35	4.13	3.65	3.21	3.19
	**No. of Medical Oncologist Visits**	3.56	2.65	2.58	2.28	2.41
	**No. of Radiation Oncologist Visits**	3.24	1.11	0.84	0.74	0.56
	**No. of General/Family Practitioner Visits**	0.22	0.13	0.05	0.06	0.06
	**No. of Other Cancer Physician Visits**	0.32	0.24	0.17	0.13	0.17
**Non-cancer Practitioner Visits**	**No. of Encounters**	32.46	23.83	20.56	18.4	19.42
	**No. of Claims**	64.7	42.79	35.87	32.86	33.4

	**Final Claims Assessment Amount**	6623.45	3376.97	2793.49	2585.1	2770.37
**Radiation Therapy**	**No. of Days of Therapy**	13.65	1.68	1.21	0.99	0.39
**Chemotherapy Cycles**	**No. of Cycles**	2.98	1.39	1.23	1.22	1

Table S4. Mean healthcare resource utilization per patient per year, calculated from diagnosis, among eNSCLC patients who received systemic therapy.

**Construct**	**Outcome**	**Year 1 (n=1210)**	**Year 2 (n=986)**	**Year 3 (n=674)**	**Year 4 (n=470)**	**Year 5 (n=318)**
**Hospitalizations**	**No. of Hospitalizations**	1.47	0.67	0.48	0.4	0.36
	**No. of Days Hospitalized**	11.16	7.5	5.63	4.19	3.75
**Ambulatory Care Services**	**No. of Encounters**	13.29	7.5	6.45	5.54	5.22
	**No. of Emergency Encounters**	2.33	1.61	1.21	0.94	0.88
	**No. of Non-emergency Encounters**	10.96	5.88	5.24	4.6	4.34
**Cancer Physician Visits**	**No. of Visits**	12.06	4.93	3.84	3.31	2.82
	**No. of Medical Oncologist Visits**	7.4	3.42	2.88	2.39	2.13
	**No. of Radiation Oncologist Visits**	3.74	1.07	0.77	0.72	0.55
	**No. of General/Family Practitioner Visits**	0.39	0.16	0.05	0.06	0.04
	**No. of Other Cancer Physician Visits**	0.52	0.28	0.13	0.14	0.1
**Non-cancer Practitioner Visits**	**No. of Encounters**	31.49	21.98	19.32	17.5	18.87

	**No. of Claims**	65.68	39.82	33.69	30.61	32.43
	**Final Claims Assessment Amount**	8162.62	3340.85	2692.73	2403.21	2578.78
**Radiation Therapy**	**No. of Days of Therapy**	18.63	1.73	1.23	1.19	0.55
**Chemotherapy Cycles**	**No. of Cycles**	7.2	1.81	1.37	1.37	0.97

Table S5. Mean healthcare resource utilization per patient per year, calculated from diagnosis, among eNSCLC patients who did not receive systemic therapy.

**Construct**	**Outcome**	**Year 1 (n=3916)**	**Year 2 (n=2538)**	**Year 3 (n=1723)**	**Year 4 (n=1198)**	**Year 5 (n=811)**
**Hospitalizations**	**No. of Hospitalizations**	1.36	0.68	0.52	0.53	0.5
	**No. of Days Hospitalized**	14.25	8.56	6.5	5.67	5.51
**Ambulatory Care Services**	**No. of Encounters**	11.46	7.61	7.11	6.8	6.52
	**No. of Emergency Encounters**	1.87	1.52	1.41	1.3	1.36
	**No. of Non-emergency Encounters**	9.59	6.1	5.7	5.5	5.15
**Cancer Physician Visits**	**No. of Visits**	2.4	1.67	1.53	1.38	1.3
	**No. of Medical Oncologist Visits**	0.58	0.75	0.78	0.73	0.77
	**No. of Radiation Oncologist Visits**	1.59	0.75	0.58	0.52	0.41
	**No. of General/Family Practitioner Visits**	0.06	0.05	0.06	0.03	0.02

	**No. of Other Cancer Physician Visits**	0.17	0.12	0.11	0.1	0.09
**Non-cancer Practitioner Visits**	**No. of Encounters**	34.28	24.66	22.32	20.91	21.54
	**No. of Claims**	68.86	42.62	38.26	36.38	37.22
	**Final Claims Assessment Amount**	7675.79	3501.43	3006.84	2843.26	2940.19
**Radiation Therapy**	**No. of Days of Therapy**	4.67	0.97	0.79	0.62	0.65
**Chemotherapy Cycles**	**No. of Cycles**	0.03	0.38	0.43	0.4	0.45
